# Supplementary material for: Iterative improvement in the automatic modular design of robot swarms
Source: PeerJ Comput Sci. 2020 Dec 7;6:e322. doi: 10.7717/peerj-cs.322 (PMC7924708; doi:10.7717/peerj-cs.322)
Supplement: Supplemental Information 3 [file peerj-cs-06-322-s003.zip › argos3/doc/api/standalone/a00564_source.html]

ARGoS: plugins/simulator/entities/box\_entity.cpp Source File


- Main Page
- Related Pages
- Namespaces
- Classes
- Files

- File List
- File Members

# plugins/simulator/entities/box\_entity.cpp

Go to the documentation of this file.

```
00001 
00007 #include "box_entity.h"
00008 #include <argos3/core/utility/math/matrix/rotationmatrix3.h>
00009 #include <argos3/core/simulator/space/space.h>
00010 #include <argos3/core/simulator/simulator.h>
00011 #include <argos3/plugins/simulator/media/led_medium.h>
00012 
00013 namespace argos {
00014 
00015    /****************************************/
00016    /****************************************/
00017 
00018    CBoxEntity::CBoxEntity():
00019       CComposableEntity(NULL),
00020       m_pcEmbodiedEntity(NULL),
00021       m_pcLEDEquippedEntity(NULL),
00022       m_fMass(1.0f),
00023       m_pcLEDMedium(NULL) {}
00024 
00025    /****************************************/
00026    /****************************************/
00027 
00028    CBoxEntity::CBoxEntity(const std::string& str_id,
00029                           const CVector3& c_position,
00030                           const CQuaternion& c_orientation,
00031                           bool b_movable,
00032                           const CVector3& c_size,
00033                           Real f_mass) :
00034       CComposableEntity(NULL, str_id),
00035       m_pcEmbodiedEntity(
00036          new CEmbodiedEntity(this,
00037                              "body_0",
00038                              c_position,
00039                              c_orientation,
00040                              b_movable)),
00041       m_pcLEDEquippedEntity(
00042          new CLEDEquippedEntity(this,
00043                                 "leds_0")),
00044       m_cSize(c_size),
00045       m_fMass(f_mass) {
00046       AddComponent(*m_pcEmbodiedEntity);
00047       AddComponent(*m_pcLEDEquippedEntity);
00048    }
00049 
00050    /****************************************/
00051    /****************************************/
00052 
00053    void CBoxEntity::Init(TConfigurationNode& t_tree) {
00054       try {
00055          /* Init parent */
00056          CComposableEntity::Init(t_tree);
00057          /* Parse XML to get the size */
00058          GetNodeAttribute(t_tree, "size", m_cSize);
00059          /* Parse XML to get the movable attribute */         
00060          bool bMovable;
00061          GetNodeAttribute(t_tree, "movable", bMovable);
00062          if(bMovable) {
00063             /* Parse XML to get the mass */
00064             GetNodeAttribute(t_tree, "mass", m_fMass);
00065          }
00066          else {
00067             m_fMass = 0.0f;
00068          }
00069          /* Create embodied entity using parsed data */
00070          m_pcEmbodiedEntity = new CEmbodiedEntity(this);
00071          AddComponent(*m_pcEmbodiedEntity);
00072          m_pcEmbodiedEntity->Init(GetNode(t_tree, "body"));
00073          m_pcEmbodiedEntity->SetMovable(bMovable);
00074          /* Init LED equipped entity component */
00075          m_pcLEDEquippedEntity = new CLEDEquippedEntity(this);
00076          AddComponent(*m_pcLEDEquippedEntity);
00077          if(NodeExists(t_tree, "leds")) {
00078             /* Create LED equipped entity
00079              * NOTE: the LEDs are not added to the medium yet
00080              */
00081             m_pcLEDEquippedEntity->Init(GetNode(t_tree, "leds"));
00082             /* Add the LEDs to the medium */
00083             std::string strMedium;
00084             GetNodeAttribute(GetNode(t_tree, "leds"), "medium", strMedium);
00085             m_pcLEDMedium = &CSimulator::GetInstance().GetMedium<CLEDMedium>(strMedium);
00086             m_pcLEDEquippedEntity->AddToMedium(*m_pcLEDMedium);
00087          }
00088          UpdateComponents();
00089       }
00090       catch(CARGoSException& ex) {
00091          THROW_ARGOSEXCEPTION_NESTED("Failed to initialize box entity \"" << GetId() << "\".", ex);
00092       }
00093    }
00094 
00095    /****************************************/
00096    /****************************************/
00097 
00098    void CBoxEntity::Reset() {
00099       /* Reset all components */
00100       CComposableEntity::Reset();
00101       /* Update components */
00102       UpdateComponents();
00103    }
00104 
00105    /****************************************/
00106    /****************************************/
00107 
00108    void CBoxEntity::EnableLEDs(CLEDMedium& c_medium) {
00109       m_pcLEDEquippedEntity->AddToMedium(c_medium);
00110    }
00111 
00112    /****************************************/
00113    /****************************************/
00114 
00115    void CBoxEntity::DisableLEDs() {
00116       m_pcLEDEquippedEntity->RemoveFromMedium();
00117    }
00118    
00119    /****************************************/
00120    /****************************************/
00121 
00122    void CBoxEntity::AddLED(const CVector3& c_offset,
00123                            const CColor& c_color) {
00124       m_pcLEDEquippedEntity->AddLED(c_offset,
00125                                     GetEmbodiedEntity().GetOriginAnchor(),
00126                                     c_color);
00127       UpdateComponents();
00128    }
00129 
00130    /****************************************/
00131    /****************************************/
00132 
00133    REGISTER_ENTITY(CBoxEntity,
00134                    "box",
00135                    "Carlo Pinciroli [ilpincy@gmail.com]",
00136                    "1.0",
00137                    "A stretchable 3D box.",
00138                    "The box entity can be used to model walls, obstacles or box-shaped grippable\n"
00139                    "objects. It can be movable or not. A movable object can be pushed and gripped.\n"
00140                    "An unmovable object is pretty much like a wall.\n\n"
00141                    "REQUIRED XML CONFIGURATION\n\n"
00142                    "To declare an unmovable object (i.e., a wall) you need the following:\n\n"
00143                    "  <arena ...>\n"
00144                    "    ...\n"
00145                    "    <box id=\"box1\" size=\"0.75,0.1,0.5\" movable=\"false\">\n"
00146                    "      <body position=\"0.4,2.3,0\" orientation=\"45,0,0\" />\n"
00147                    "    </box>\n"
00148                    "    ...\n"
00149                    "  </arena>\n\n"
00150                    "To declare a movable object you need the following:\n\n"
00151                    "  <arena ...>\n"
00152                    "    ...\n"
00153                    "    <box id=\"box1\" size=\"0.75,0.1,0.5\" movable=\"true\" mass=\"2.5\">\n"
00154                    "      <body position=\"0.4,2.3,0\" orientation=\"45,0,0\" />\n"
00155                    "    </box>\n"
00156                    "    ...\n"
00157                    "  </arena>\n\n"
00158                    "The 'id' attribute is necessary and must be unique among the entities. If two\n"
00159                    "entities share the same id, initialization aborts.\n"
00160                    "The 'size' attribute specifies the size of the box along the three axes, in\n"
00161                    "the X,Y,Z order. When you add a box, imagine it initially unrotated and\n"
00162                    "centered in the origin. The size, then, corresponds to the extent along the X,\n"
00163                    "Y and Z axes.\n"
00164                    "The 'movable' attribute specifies whether or not the object is movable. When\n"
00165                    "set to 'false', the object is unmovable: if another object pushes against it,\n"
00166                    "the box won't move. When the attribute is set to 'true', the box is movable\n"
00167                    "upon pushing or gripping. When an object is movable, the 'mass' attribute is\n"
00168                    "required.\n"
00169                    "The 'mass' attribute quantifies the mass of the box in kg.\n"
00170                    "The 'body/position' attribute specifies the position of the base of the box in\n"
00171                    "the arena. The three values are in the X,Y,Z order.\n"
00172                    "The 'body/orientation' attribute specifies the orientation of the 3D box. All\n"
00173                    "rotations are performed with respect to the center of mass. The order of the\n"
00174                    "angles is Z,Y,X, which means that the first number corresponds to the rotation\n"
00175                    "around the Z axis, the second around Y and the last around X. This reflects\n"
00176                    "the internal convention used in ARGoS, in which rotations are performed in\n"
00177                    "that order. Angles are expressed in degrees.\n\n"
00178                    "OPTIONAL XML CONFIGURATION\n\n"
00179                    "It is possible to add any number of colored LEDs to the box. In this way,\n"
00180                    "the box is visible with a robot camera. The position and color of the\n"
00181                    "LEDs is specified with the following syntax:\n\n"
00182                    "  <arena ...>\n"
00183                    "    ...\n"
00184                    "    <box id=\"box1\" size=\"0.75,0.1,0.5\" movable=\"true\" mass=\"2.5\">\n"
00185                    "      <body position=\"0.4,2.3,0\" orientation=\"45,0,0\" />\n"
00186                    "      <leds medium=\"id_of_led_medium\">\n"
00187                    "        <led offset=\" 0.15, 0.15,0.15\" anchor=\"origin\" color=\"white\" />\n"
00188                    "        <led offset=\"-0.15, 0.15,0\"    anchor=\"origin\" color=\"red\"   />\n"
00189                    "        <led offset=\" 0.15, 0.15,0\"    anchor=\"origin\" color=\"blue\"  />\n"
00190                    "        <led offset=\" 0.15,-0.15,0\"    anchor=\"origin\" color=\"green\" />\n"
00191                    "      </leds>\n"
00192                    "    </box>\n"
00193                    "    ...\n"
00194                    "  </arena>\n\n"
00195                    "In the example, four LEDs are added to the box. The LEDs have\n"
00196                    "different colors and are located one on the top and three\n"
00197                    "around the box. The LEDs are managed by the LED medium declared in\n"
00198                    "the <media> section of the configuration file with id \"id_of_led_medium\"",
00199                    "Usable"
00200       );
00201 
00202    /****************************************/
00203    /****************************************/
00204 
00205    REGISTER_STANDARD_SPACE_OPERATIONS_ON_COMPOSABLE(CBoxEntity);
00206 
00207    /****************************************/
00208    /****************************************/
00209 
00210 }
```

---

Generated on 10 Jul 2018 for ARGoS by 
 1.6.1
